# 8,9-Isopropyl­idenedi­oxy-3-*p*-tolyl-1,6-dioxa-3-aza­spiro­[4.5]decane-2,10-dione

**DOI:** 10.1107/S1600536808034259

**Published:** 2008-10-25

**Authors:** Chun-Sheng Ling, Qiang Wu, Shan-Shan Li

**Affiliations:** a Pharmaceutical College of Henan University, Kaifeng 475004, People’s Republic of China; b Institute of Pharmacy, Henan University, Kaifeng 475004, People’s Republic of China; c College of Chemistry and Environmental Engineering, Beijing Technology and Business University, Beijing 100037, People’s Republic of China

## Abstract

In the title compound, C_17_H_19_NO_6_, which may serve as a ketone catalyst for the asymmetric epoxidation of olefins, the crystal packing is consolidated by C—H⋯O inter­actions.

## Related literature

For general background, see: Denmark & Wu (1999 ▶); Shi (2004 ▶); Yang (2004 ▶). For the synthesis, see: Zhao *et al.* (2006 ▶).
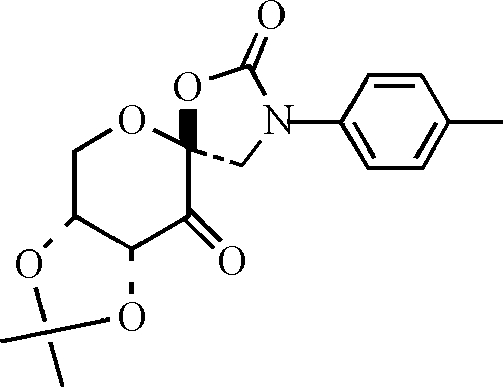


## Experimental

### 

#### Crystal data

C_17_H_19_NO_6_
*M* *_r_* = 333.33
Monoclinic, 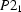

*a* = 11.1268 (8) Å
*b* = 6.3163 (5) Å
*c* = 11.8697 (8) Å
β = 94.084 (1)°
*V* = 832.09 (11) Å^3^
*Z* = 2
Mo *K*α radiation
μ = 0.10 mm^−1^
*T* = 296 (2) K
0.18 × 0.15 × 0.13 mm

#### Data collection

Bruker SMART CCD diffractometer
Absorption correction: multi-scan (*SADABS*; Sheldrick, 2001 ▶) *T*_min_ = 0.982, *T*_max_ = 0.987
8821 measured reflections
1795 independent reflections
1389 reflections with *I* > 2σ(*I*)
*R*_int_ = 0.028

#### Refinement

*R*[*F* ^2^ > 2σ(*F* ^2^)] = 0.034
*wR*(*F* ^2^) = 0.075
*S* = 1.06
1795 reflections
220 parameters
1 restraint
H-atom parameters constrained
Δρ_max_ = 0.10 e Å^−3^
Δρ_min_ = −0.13 e Å^−3^

### 

Data collection: *SMART* (Bruker, 2001 ▶); cell refinement: *SAINT-Plus* (Bruker, 2001 ▶); data reduction: *SAINT-Plus*; program(s) used to solve structure: *SHELXS97* (Sheldrick, 2008 ▶); program(s) used to refine structure: *SHELXL97* (Sheldrick, 2008 ▶); molecular graphics: *PLATON* (Spek, 2003 ▶); software used to prepare material for publication: *PLATON*.

## Supplementary Material

Crystal structure: contains datablocks global, I. DOI: 10.1107/S1600536808034259/hb2819sup1.cif

Structure factors: contains datablocks I. DOI: 10.1107/S1600536808034259/hb2819Isup2.hkl

Additional supplementary materials:  crystallographic information; 3D view; checkCIF report

## Figures and Tables

**Table 1:** Hydrogen-bond geometry (Å, °)

*D*—H⋯*A*	*D*—H	H⋯*A*	*D*⋯*A*	*D*—H⋯*A*
C9—H9*B*⋯O1^i^	0.97	2.54	3.093 (3)	116
C14—H14*B*⋯O4^ii^	0.97	2.55	3.426 (3)	151

Symmetry codes: (i) 
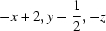
; (ii) 
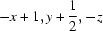
.

**Fig. 1.**
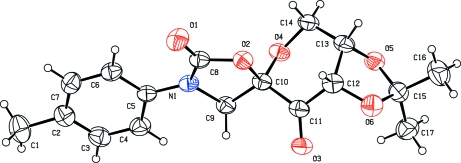
The molecular structure of (I) with displacement ellipsoids for the non-hydrogen atoms drawn at the 50% probability level.

**Fig. 2.**
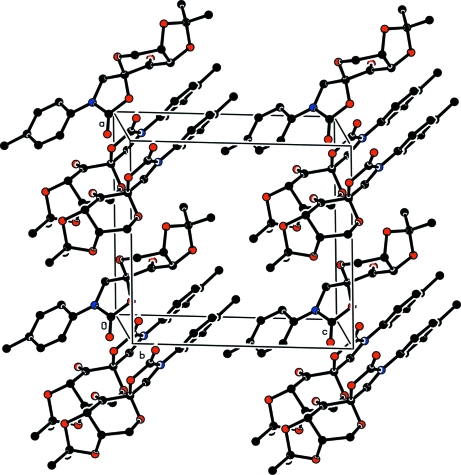
Packing of the title compound (I), H atoms are omitted for clarity.

**Fig. 3.**
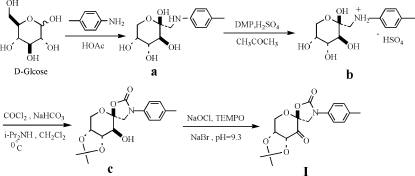
The formation of the title compound.

## supplementary crystallographic information

### Comment

Dioxiranes generated *in situ* from chiral ketones are effective for the asymmetric epoxidation of olefins (Denmark & Wu, 1999; Shi, 2004; Yang, 2004).  As part of our own studies in this area, we now report the synthesis and structure of the title compound, (I).

The compound (I) consists of a four-ring system, including a phenyl ring, a pyran ring, a dioxolane ring and an oxazolidine ring, and which displays a chair molecular framework (Fig. 1).  In the structure of (I), the S(6) ring of O4/C10/C11/C12/C13/C14 is nonplanar, charactrtized by a O4–C10–C11–C12 torsion angle of 61.1 (2) °.  The stereogenic centres C10, C12 and C13 were assigned R, S, and S configurations, respectively.

In the crystal, some short C—H···O interactions (Table 1) may help to establish the packing (Fig. 2).

### Experimental

The title compound was made by the method of Zhao *et al.* (2006), starting from D-glucose and 4-methyl-benzenamine to yield colorless blocks of (I).  The molecular formula, C_17_H_19_NO_6_, was established by ESI-MS, m/*z*: 356(*M*+Na), 334(*M*+H), 232, 204, 108. Spectroscopic analysis, 1H NMR (400 MHz, CDCl_3_): *δ*7.40 (d, *J*=5.4 Hz, 2H, ArH), 7.19 (d, *J*=5.4 Hz, 2H, ArH), 4.87 (d, *J*=5.7 Hz, 1H), 4.74 (d, *J*=10.2 Hz, 1H), 4.66–4.61 (m, 2H), 4.27 (d, *J*=13.8 Hz, 1H), 3.74 (d, *J*=10.2 Hz, 1H), 2.34 (s, 3H, ArCH_3_), 1.49 (s, 3H, –CH_3_), 1.44 (s, 3H, –CH_3_).

### Refinement

Anomalous dispersion was negligible and Firedel pairs were merged before refinement. The H atoms were gemoetrically placed (C—H = 0.93–0.98Å) and refined as riding with *U*_iso_(*H*) = 1.2*U*_eq_(C) 1.5*U*_eq_(methyl C).

### Crystal data

**Table:**

C_17_H_19_NO_6_	*F*(000) = 352
*M**_r_* = 333.33	*D*_x_ = 1.330 Mg m^−^^3^
Monoclinic, *P*2_1_	Mo *K*α radiation, λ = 0.71073 Å
Hall symbol: P 2y b	Cell parameters from 2427 reflections
*a* = 11.1268 (8) Å	θ = 2.4–21.1°
*b* = 6.3163 (5) Å	µ = 0.10 mm^−^^1^
*c* = 11.8697 (8) Å	*T* = 296 K
β = 94.084 (1)°	Block, colorless
*V* = 832.09 (11) Å^3^	0.18 × 0.15 × 0.13 mm
*Z* = 2	

### Data collection

**Table:**

Bruker SMART CCD diffractometer	1795 independent reflections
Radiation source: fine-focus sealed tube	1389 reflections with *I* > 2σ(*I*)
graphite	*R*_int_ = 0.028
ω scans	θ_max_ = 26.0°, θ_min_ = 1.7°
Absorption correction: multi-scan (SADABS; Sheldrick, 2001)	*h* = −13→13
*T*_min_ = 0.982, *T*_max_ = 0.987	*k* = −7→7
8821 measured reflections	*l* = −14→14

### Refinement

**Table:**

Refinement on *F*^2^	Primary atom site location: structure-invariant direct methods
Least-squares matrix: full	Secondary atom site location: difference Fourier map
*R*[*F*^2^ > 2σ(*F*^2^)] = 0.034	Hydrogen site location: inferred from neighbouring sites
*wR*(*F*^2^) = 0.075	H-atom parameters constrained
*S* = 1.06	*w* = 1/[σ^2^(*F*_o_^2^) + (0.0303*P*)^2^ + 0.0894*P*] where *P* = (*F*_o_^2^ + 2*F*_c_^2^)/3
1795 reflections	(Δ/σ)_max_ < 0.001
220 parameters	Δρ_max_ = 0.10 e Å^−^^3^
1 restraint	Δρ_min_ = −0.13 e Å^−^^3^

### Special details

**Table:**

Geometry. All e.s.d.'s (except the e.s.d. in the dihedral angle between two l.s. planes) are estimated using the full covariance matrix. The cell e.s.d.'s are taken into account individually in the estimation of e.s.d.'s in distances, angles and torsion angles; correlations between e.s.d.'s in cell parameters are only used when they are defined by crystal symmetry. An approximate (isotropic) treatment of cell e.s.d.'s is used for estimating e.s.d.'s involving l.s. planes.
Refinement. Refinement of *F*^2^ against ALL reflections. The weighted *R*-factor *wR* and goodness of fit *S* are based on *F*^2^, conventional *R*-factors *R* are based on *F*, with *F* set to zero for negative *F*^2^. The threshold expression of *F*^2^ > σ(*F*^2^) is used only for calculating *R*-factors(gt) *etc*. and is not relevant to the choice of reflections for refinement. *R*-factors based on *F*^2^ are statistically about twice as large as those based on *F*, and *R*- factors based on ALL data will be even larger.

### Fractional atomic coordinates and isotropic or equivalent isotropic displacement parameters (Å^2^)

**Table:**

	*x*	*y*	*z*	*U*_iso_*/*U*_eq_	
N1	0.89223 (17)	0.9579 (3)	0.12879 (17)	0.0543 (5)	
O1	1.00555 (17)	1.2504 (3)	0.08456 (16)	0.0792 (6)	
O2	0.84070 (14)	1.1560 (3)	−0.02227 (14)	0.0588 (5)	
O3	0.70022 (18)	0.7396 (3)	−0.15295 (19)	0.0810 (6)	
O4	0.64278 (14)	1.1097 (3)	0.02397 (14)	0.0600 (5)	
O5	0.47051 (15)	1.1373 (3)	−0.18543 (15)	0.0665 (5)	
O6	0.59983 (16)	1.0190 (4)	−0.31240 (15)	0.0766 (6)	
C1	1.1700 (3)	0.5776 (7)	0.5018 (3)	0.0997 (12)	
H1A	1.1778	0.6722	0.5652	0.150*	
H1B	1.2484	0.5455	0.4775	0.150*	
H1C	1.1316	0.4492	0.5235	0.150*	
C2	1.0944 (3)	0.6815 (5)	0.4059 (2)	0.0705 (8)	
C3	1.0017 (3)	0.5773 (5)	0.3486 (2)	0.0760 (8)	
H3	0.9824	0.4413	0.3714	0.091*	
C4	0.9355 (3)	0.6666 (5)	0.2580 (2)	0.0687 (8)	
H4	0.8727	0.5907	0.2212	0.082*	
C5	0.9620 (2)	0.8692 (4)	0.2214 (2)	0.0534 (6)	
C6	1.0555 (2)	0.9785 (5)	0.2790 (2)	0.0648 (7)	
H6	1.0751	1.1145	0.2567	0.078*	
C7	1.1195 (2)	0.8837 (6)	0.3702 (2)	0.0727 (8)	
H7	1.1815	0.9590	0.4086	0.087*	
C8	0.9231 (2)	1.1294 (4)	0.0685 (2)	0.0563 (6)	
C9	0.7901 (2)	0.8463 (4)	0.0715 (2)	0.0568 (6)	
H9A	0.7294	0.8121	0.1234	0.068*	
H9B	0.8156	0.7175	0.0357	0.068*	
C10	0.74453 (19)	1.0086 (4)	−0.0148 (2)	0.0524 (6)	
C11	0.7064 (2)	0.9266 (4)	−0.1330 (2)	0.0566 (6)	
C12	0.6676 (2)	1.0984 (5)	−0.2160 (2)	0.0628 (7)	
H12	0.7381	1.1759	−0.2389	0.075*	
C13	0.5811 (2)	1.2495 (4)	−0.1640 (2)	0.0647 (7)	
H13	0.5778	1.3830	−0.2062	0.078*	
C14	0.6057 (2)	1.2941 (4)	−0.0408 (2)	0.0677 (7)	
H14A	0.6684	1.4007	−0.0314	0.081*	
H14B	0.5335	1.3517	−0.0114	0.081*	
C15	0.4747 (2)	1.0412 (5)	−0.2935 (2)	0.0696 (8)	
C16	0.4154 (3)	1.1789 (7)	−0.3854 (3)	0.1116 (13)	
H16A	0.3316	1.1956	−0.3732	0.167*	
H16B	0.4234	1.1136	−0.4576	0.167*	
H16C	0.4537	1.3152	−0.3840	0.167*	
C17	0.4167 (3)	0.8262 (6)	−0.2888 (3)	0.0914 (10)	
H17A	0.4574	0.7441	−0.2296	0.137*	
H17B	0.4222	0.7553	−0.3597	0.137*	
H17C	0.3335	0.8422	−0.2739	0.137*	

### Atomic displacement parameters (Å^2^)

**Table:**

	*U*^11^	*U*^22^	*U*^33^	*U*^12^	*U*^13^	*U*^23^
N1	0.0565 (12)	0.0435 (12)	0.0639 (12)	−0.0099 (10)	0.0112 (10)	−0.0006 (10)
O1	0.0775 (12)	0.0680 (12)	0.0918 (13)	−0.0344 (12)	0.0032 (10)	0.0086 (11)
O2	0.0532 (9)	0.0492 (10)	0.0746 (11)	−0.0102 (8)	0.0097 (8)	0.0076 (9)
O3	0.0798 (13)	0.0514 (12)	0.1092 (15)	0.0067 (11)	−0.0109 (11)	−0.0152 (12)
O4	0.0554 (9)	0.0535 (11)	0.0729 (11)	0.0052 (9)	0.0179 (8)	0.0002 (9)
O5	0.0617 (10)	0.0617 (11)	0.0763 (12)	0.0064 (10)	0.0058 (8)	−0.0065 (11)
O6	0.0769 (12)	0.0874 (14)	0.0670 (11)	0.0026 (12)	0.0163 (10)	−0.0039 (11)
C1	0.087 (2)	0.131 (3)	0.082 (2)	0.014 (2)	0.0079 (17)	0.023 (2)
C2	0.0640 (17)	0.081 (2)	0.0681 (18)	0.0086 (16)	0.0169 (14)	0.0026 (16)
C3	0.089 (2)	0.0619 (19)	0.0775 (19)	0.0032 (17)	0.0113 (16)	0.0108 (16)
C4	0.0782 (18)	0.0552 (17)	0.0727 (18)	−0.0080 (15)	0.0053 (14)	−0.0008 (16)
C5	0.0565 (14)	0.0485 (15)	0.0563 (15)	0.0007 (12)	0.0131 (12)	−0.0047 (12)
C6	0.0637 (16)	0.0599 (17)	0.0719 (17)	−0.0069 (14)	0.0128 (14)	−0.0061 (15)
C7	0.0608 (17)	0.088 (2)	0.0695 (19)	−0.0023 (17)	0.0066 (14)	−0.0095 (17)
C8	0.0560 (14)	0.0470 (15)	0.0672 (15)	−0.0056 (14)	0.0142 (12)	−0.0002 (14)
C9	0.0501 (13)	0.0438 (13)	0.0771 (16)	−0.0094 (12)	0.0082 (12)	0.0038 (13)
C10	0.0439 (12)	0.0439 (13)	0.0708 (16)	−0.0061 (12)	0.0147 (11)	0.0010 (12)
C11	0.0418 (13)	0.0497 (15)	0.0799 (18)	−0.0014 (11)	0.0168 (12)	−0.0023 (14)
C12	0.0629 (15)	0.0604 (16)	0.0671 (16)	−0.0096 (14)	0.0182 (13)	0.0037 (14)
C13	0.0712 (17)	0.0429 (14)	0.0803 (19)	0.0024 (14)	0.0080 (14)	0.0057 (15)
C14	0.0699 (17)	0.0472 (16)	0.086 (2)	0.0098 (14)	0.0052 (14)	−0.0095 (15)
C15	0.0694 (18)	0.0691 (19)	0.0700 (18)	0.0084 (15)	0.0032 (14)	0.0037 (16)
C16	0.131 (3)	0.108 (3)	0.093 (2)	0.025 (3)	−0.012 (2)	0.023 (2)
C17	0.086 (2)	0.084 (3)	0.104 (2)	−0.009 (2)	0.0007 (18)	−0.012 (2)

### Geometric parameters (Å, °)

**Table:**

N1—C8	1.356 (3)	C5—C6	1.387 (3)
N1—C5	1.415 (3)	C6—C7	1.389 (4)
N1—C9	1.463 (3)	C6—H6	0.9300
O1—C8	1.199 (3)	C7—H7	0.9300
O2—C8	1.374 (3)	C9—C10	1.511 (3)
O2—C10	1.426 (3)	C9—H9A	0.9700
O3—C11	1.205 (3)	C9—H9B	0.9700
O4—C10	1.405 (3)	C10—C11	1.528 (4)
O4—C14	1.440 (3)	C11—C12	1.508 (4)
O5—C15	1.423 (3)	C12—C13	1.517 (4)
O5—C13	1.427 (3)	C12—H12	0.9800
O6—C12	1.417 (3)	C13—C14	1.495 (4)
O6—C15	1.432 (3)	C13—H13	0.9800
C1—C2	1.516 (4)	C14—H14A	0.9700
C1—H1A	0.9600	C14—H14B	0.9700
C1—H1B	0.9600	C15—C17	1.506 (4)
C1—H1C	0.9600	C15—C16	1.510 (4)
C2—C3	1.364 (4)	C16—H16A	0.9600
C2—C7	1.380 (5)	C16—H16B	0.9600
C3—C4	1.380 (4)	C16—H16C	0.9600
C3—H3	0.9300	C17—H17A	0.9600
C4—C5	1.390 (4)	C17—H17B	0.9600
C4—H4	0.9300	C17—H17C	0.9600
			
C8—N1—C5	125.5 (2)	O4—C10—C11	106.10 (18)
C8—N1—C9	110.9 (2)	O2—C10—C11	108.94 (19)
C5—N1—C9	122.3 (2)	C9—C10—C11	116.8 (2)
C8—O2—C10	109.46 (18)	O3—C11—C12	124.6 (3)
C10—O4—C14	113.54 (19)	O3—C11—C10	121.4 (3)
C15—O5—C13	106.81 (19)	C12—C11—C10	113.8 (2)
C12—O6—C15	107.8 (2)	O6—C12—C11	112.6 (2)
C2—C1—H1A	109.5	O6—C12—C13	103.6 (2)
C2—C1—H1B	109.5	C11—C12—C13	110.3 (2)
H1A—C1—H1B	109.5	O6—C12—H12	110.1
C2—C1—H1C	109.5	C11—C12—H12	110.1
H1A—C1—H1C	109.5	C13—C12—H12	110.1
H1B—C1—H1C	109.5	O5—C13—C14	111.3 (2)
C3—C2—C7	117.1 (3)	O5—C13—C12	100.3 (2)
C3—C2—C1	121.7 (3)	C14—C13—C12	116.0 (2)
C7—C2—C1	121.2 (3)	O5—C13—H13	109.6
C2—C3—C4	122.3 (3)	C14—C13—H13	109.6
C2—C3—H3	118.9	C12—C13—H13	109.6
C4—C3—H3	118.9	O4—C14—C13	113.3 (2)
C3—C4—C5	120.4 (3)	O4—C14—H14A	108.9
C3—C4—H4	119.8	C13—C14—H14A	108.9
C5—C4—H4	119.8	O4—C14—H14B	108.9
C6—C5—C4	118.2 (3)	C13—C14—H14B	108.9
C6—C5—N1	122.4 (2)	H14A—C14—H14B	107.7
C4—C5—N1	119.4 (2)	O5—C15—O6	106.1 (2)
C5—C6—C7	119.7 (3)	O5—C15—C17	108.0 (3)
C5—C6—H6	120.2	O6—C15—C17	110.0 (2)
C7—C6—H6	120.2	O5—C15—C16	111.4 (3)
C2—C7—C6	122.3 (3)	O6—C15—C16	108.8 (3)
C2—C7—H7	118.9	C17—C15—C16	112.3 (3)
C6—C7—H7	118.9	C15—C16—H16A	109.5
O1—C8—N1	130.1 (2)	C15—C16—H16B	109.5
O1—C8—O2	120.5 (2)	H16A—C16—H16B	109.5
N1—C8—O2	109.4 (2)	C15—C16—H16C	109.5
N1—C9—C10	101.55 (19)	H16A—C16—H16C	109.5
N1—C9—H9A	111.5	H16B—C16—H16C	109.5
C10—C9—H9A	111.5	C15—C17—H17A	109.5
N1—C9—H9B	111.5	C15—C17—H17B	109.5
C10—C9—H9B	111.5	H17A—C17—H17B	109.5
H9A—C9—H9B	109.3	C15—C17—H17C	109.5
O4—C10—O2	110.5 (2)	H17A—C17—H17C	109.5
O4—C10—C9	109.00 (19)	H17B—C17—H17C	109.5
O2—C10—C9	105.54 (17)		
			
C7—C2—C3—C4	0.6 (4)	N1—C9—C10—O2	17.4 (2)
C1—C2—C3—C4	−177.1 (3)	N1—C9—C10—C11	138.56 (19)
C2—C3—C4—C5	0.2 (4)	O4—C10—C11—O3	−113.6 (3)
C3—C4—C5—C6	−0.6 (4)	O2—C10—C11—O3	127.4 (3)
C3—C4—C5—N1	−179.6 (2)	C9—C10—C11—O3	8.0 (3)
C8—N1—C5—C6	16.7 (4)	O4—C10—C11—C12	61.1 (2)
C9—N1—C5—C6	−177.0 (2)	O2—C10—C11—C12	−57.9 (2)
C8—N1—C5—C4	−164.3 (2)	C9—C10—C11—C12	−177.25 (19)
C9—N1—C5—C4	1.9 (3)	C15—O6—C12—C11	95.7 (3)
C4—C5—C6—C7	0.2 (4)	C15—O6—C12—C13	−23.5 (3)
N1—C5—C6—C7	179.2 (2)	O3—C11—C12—O6	12.3 (4)
C3—C2—C7—C6	−1.0 (4)	C10—C11—C12—O6	−162.21 (18)
C1—C2—C7—C6	176.7 (3)	O3—C11—C12—C13	127.5 (3)
C5—C6—C7—C2	0.6 (4)	C10—C11—C12—C13	−47.0 (3)
C5—N1—C8—O1	−7.5 (4)	C15—O5—C13—C14	−160.6 (2)
C9—N1—C8—O1	−175.0 (3)	C15—O5—C13—C12	−37.3 (2)
C5—N1—C8—O2	173.46 (19)	O6—C12—C13—O5	36.9 (2)
C9—N1—C8—O2	5.9 (3)	C11—C12—C13—O5	−83.8 (2)
C10—O2—C8—O1	−172.9 (2)	O6—C12—C13—C14	157.0 (2)
C10—O2—C8—N1	6.2 (3)	C11—C12—C13—C14	36.3 (3)
C8—N1—C9—C10	−14.5 (2)	C10—O4—C14—C13	56.3 (3)
C5—N1—C9—C10	177.48 (19)	O5—C13—C14—O4	73.7 (3)
C14—O4—C10—O2	53.3 (3)	C12—C13—C14—O4	−40.2 (3)
C14—O4—C10—C9	168.83 (19)	C13—O5—C15—O6	24.2 (3)
C14—O4—C10—C11	−64.6 (2)	C13—O5—C15—C17	142.0 (2)
C8—O2—C10—O4	102.5 (2)	C13—O5—C15—C16	−94.1 (3)
C8—O2—C10—C9	−15.2 (3)	C12—O6—C15—O5	0.8 (3)
C8—O2—C10—C11	−141.4 (2)	C12—O6—C15—C17	−115.8 (3)
N1—C9—C10—O4	−101.3 (2)	C12—O6—C15—C16	120.8 (3)

### Hydrogen-bond geometry (Å, °)

**Table:**

*D*—H···*A*	*D*—H	H···*A*	*D*···*A*	*D*—H···*A*
C9—H9B···O1^i^	0.97	2.54	3.093 (3)	116
C14—H14B···O4^ii^	0.97	2.55	3.426 (3)	151

Symmetry codes: (i) −*x*+2, *y*−1/2, −*z*; (ii) −*x*+1, *y*+1/2, −*z*.
